# Induction of Innate Immune Response by TLR3 Agonist Protects Mice against SARS-CoV-2 Infection

**DOI:** 10.3390/v14020189

**Published:** 2022-01-19

**Authors:** Hadas Tamir, Sharon Melamed, Noam Erez, Boaz Politi, Yfat Yahalom-Ronen, Hagit Achdout, Shlomi Lazar, Hila Gutman, Roy Avraham, Shay Weiss, Nir Paran, Tomer Israely

**Affiliations:** 1 Department of Infectious Diseases, Israel Institute for Biological Research, P.O. Box 19, Ness Ziona 7410001, Israel; hadast@iibr.gov.il (H.T.); sharonm@iibr.gov.il (S.M.); noame@iibr.gov.il (N.E.); boazp@iibr.gov.il (B.P.); yfatyr@iibr.gov.il (Y.Y.-R.); hagita@iibr.gov.il (H.A.); roya@iibr.gov.il (R.A.); shayw@iibr.gov.il (S.W.); nirp@iibr.gov.il (N.P.); 2 Department of Pharmacology, Israel Institute for Biological Research, P.O. Box 19, Ness Ziona 7410001, Israel; shlomil@iibr.gov.il (S.L.); hilag@iibr.gov.il (H.G.)

**Keywords:** SARS-CoV-2, COVID-19, hACE2-K18 transgenic mice, TLR3, Poly(I:C), innate immune response

## Abstract

SARS-CoV-2, a member of the coronavirus family, is the causative agent of the COVID-19 pandemic. Currently, there is still an urgent need in developing an efficient therapeutic intervention. In this study, we aimed at evaluating the therapeutic effect of a single intranasal treatment of the TLR3/MDA5 synthetic agonist Poly(I:C) against a lethal dose of SARS-CoV-2 in K18-hACE2 transgenic mice. We demonstrate here that early Poly(I:C) treatment acts synergistically with SARS-CoV-2 to induce an intense, immediate and transient upregulation of innate immunity-related genes in lungs. This effect is accompanied by viral load reduction, lung and brain cytokine storms prevention and increased levels of macrophages and NK cells, resulting in 83% mice survival, concomitantly with long-term immunization. Thus, priming the lung innate immunity by Poly(I:C) or alike may provide an immediate, efficient and safe protective measure against SARS-CoV-2 infection.

## 1. Introduction

The coronavirus disease 19 (COVID-19) pandemic imposes unprecedented efforts in developing efficient and safe vaccines and therapeutics, within a very short period. While several vaccine strategies have already been approved by the Food and Drug Administration (FDA) via a rapid procedure, efficient therapeutic solutions for COVID-19 patients are still required. Several strategies have been promoted as potential therapeutic interventions [1] and can be divided into two major categories: targeting the virus itself and manipulating host signaling response [1]. The first category includes interfering with SARS-CoV-2 binding to its receptor angiotensin-converting enzyme 2 (ACE2) by monoclonal antibodies (mAbs) [2,3,4,5], inhibiting membrane fusion by protease inhibitors [6] or inhibiting viral replication [7,8]. The second category is primarily aimed at modulating the immune system either by activating the innate immune response or by preventing or reducing the hyperimmune cytokine storm [9]. Following infection, the innate immune system detects viral invasion by a specialized and diverse group of cytoplasmic or membrane-bound pattern recognition receptors (PRRs) [10]. Membrane-bound PRRs include toll-like receptors (TLRs) and C-type lectin receptors (CLRs). TLRs are located at the outer cell membranes or endosomes, recognize specific bacterial glycolipids, lipopolysaccharides, single- and double-strand viral RNA and bacterial and viral DNA. CLRs recognize bacteria, viruses and parasites by their typical carbohydrates. Cytoplasmic PRRs include RIG-1-like receptors (RLRs) and nucleotide oligomerization domain (NOD)-like receptors (NLRs). The major receptors involved in viral components recognition include RIG-1, MDA-5, NLRs and TLRs. Signal transduction following receptor activation is mediated by adaptor molecules such as MyD88, TRIF, MAVS or STING that trigger intracellular cascades via IRF3, IRF7 and IKK, leading to the activation of type I interferon (IFN I) response and secretion of inflammatory cytokines [11]. IFN I upregulation is probably the most powerful innate immune line of defense against viral invasion. It induces hundreds of antiviral interferon-stimulated genes (ISG) [12], cytokine release and recruitment of inflammatory cells (such as macrophages, NK cells and neutrophils) to the site of infection.

COVID-19 is associated with a delayed IFN I response, allowing rapid virus replication in lung type II pneumocytes, cytokines and chemokine induction and inflammatory monocyte–macrophage (IMM) recruitment to the lungs, which results in morbidity and mortality [13]. In view of the central antiviral role of IFNs, it is not surprising that SARS-CoV-2 adopted a strategy of hijacking the translation machinery of the cell by inhibiting antiviral defense pathways, including IFN-mediated signaling [14]. This immune evasion strategy is shared by many other viruses such as vaccinia, hepatitis B and influenza [15]. This is done by direct inhibition of IFN production, interferon-mediated intracellular signaling disruption or interference with antiviral protein functions [15,16]. It has been suggested that the delayed innate immune response, induced by SARS-CoV-2, provides a “time window” for viral replication, leading eventually to cytokine storm and death [17]. It was also shown that in the case of other corona family members, namely SARS and MERS, the administration of exogenous Type I IFNs reduced the disease’s severe symptoms [17], offering a promising therapeutic option for COVID-19 as well [18].

Poly(I:C) is a synthetic analog of double-strand RNA (dsRNA) with an immunostimulatory effect following binding to TLR3 and MDA5 PRRs [19]. This synthetic PRR agonist is approved for human use (https://clinicaltrials.gov/ct2/show/NCT02532413 (accessed on 12 January 2022)); [20]. The application of Poly(I:C) in tumor immunotherapy as a vaccine adjuvant has been extensively investigated [21]. Moreover, the positive effect of Poly(I:C) has also been demonstrated against a variety of viruses such as herpes simplex virus type 1 [22], Ebola [23], chikungunya [24], SARS-CoV [20], hepatitis B [25] and ectromelia [26].

We hypothesized that Poly(I:C), which has been shown to induce IFN I and inflammatory cytokine expression [27], may prime the innate immune system of the lungs, thus enabling a rapid onset of the innate immune response against SARS-CoV-2 infection in K18-hACE2 transgenic mice.

The K18-hACE2 animal model was selected in view of its ability to recapitulate key aspects of COVID-19, from mild through severe symptoms, and death. In the present study, we demonstrated that SARS-CoV-2 induces a delayed, robust cytokine storm in untreated mice lungs and brains, which eventually succumb to infection. This observed cytokine storm could be prevented by intranasal (i.n.) Poly(I:C) treatment immediately following infection, enabling a rapid and transient upregulation of innate inflammatory-related genes, viral load reduction, alleviation of lung damage, resulting in mice protection. 

## 2. Methods

### 2.1. Cells

Vero E6 cells (ATCC^®^ CRL-1586^TM^) cells were maintained in Dulbecco’s modified Eagle’s medium (DMEM) supplemented with 10% fetal bovine serum (FBS), MEM nonessential amino acids, 2 nM L-glutamine, 100 units/mL penicillin, 0.1 mg/mL streptomycin and 12.5 units/mL nystatin (Biological Industries, Beit-Haemek, Israel). Cells were cultured at 37 °C in a 5% CO_2_ and 95% air atmosphere.

### 2.2. Viruses

The SARS-CoV-2 isolate Human 2019-nCoV ex China strain BavPat1/2020 was kindly provided by Prof. Dr. Christian Drosten (Charité, Berlin, Germany) through the European Virus Archive Global (EVAg Ref-SKU: 026 V-03883). The viral stocks were propagated (4 passages) and titrated in Vero E6 cells. The genome sequence has been deposited at the GISAID EpiCoV coronavirus SARS-CoV-2 platform database under the identifier EPI_ISL_3838266.

### 2.3. Animal Experiments

All animal experiments involving SARS-CoV-2 were conducted in a biosafety level 3 (BSL3) facility. Treatment of animals was in accordance with regulations outlined in the U.S. Department of Agriculture (USDA) Animal Welfare Act and the conditions specified in the Guide for Care and Use of Laboratory Animals [28]. Animal studies were approved by the local Israel Institute for Biological Research. Institutional Animal Care and Use Committee and experimental procedures were performed under Protocol Numbers M-61-20 and M-03-21. Female K18-hACE2 transgenic mice (The Jackson Laboratory (Bar Harbor, ME, USA)) 6–8 weeks old were maintained at 20−22 °C and a relative humidity of 50 ± 10% on a 12 h light/dark cycle. Animals were fed with commercial rodent chow (Koffolk Inc., Neot Hovav, Israel) and provided with tap water ad libitum. Prior to infection, mice were kept in groups of 10. Mice were randomly assigned to experimental groups.

The virus was diluted in phosphate-buffered saline (PBS) supplemented with 2% FBS (Biological Industries, Beit-Haemek, Israel). Anesthetized animals (75 mg/kg ketamine, 7.5 mg/kg xylazine in PBS) were infected by 20 µL i.n. instillation of 500 or 2000 PFU/mouse.

Poly(I:C) (high molecular weight, InvivoGen, San Diego, CA, USA) treatment: K18-hACE2 transgenic mice were treated intranasally with 1.5 mg/kg either 6 h before infection or 0, 6 or 12 h post infection. This dose was selected based on previous reports [20,29,30].

### 2.4. Determination of Viral Load in Organs

Viral loads were determined 2 and 5 days post SARS-CoV-2 infection. Lungs, nasal turbinates and brains were harvested and stored at −80 °C until further processing. Organs were processed for titration in 1.5 mL of ice-cold PBS as previously described [31]. Part of the processed tissue samples was used immediately for RNA extraction for gene expression, while the other part was kept at −80 °C until further processing for viral titration.

SARS-CoV-2 viral load was determined using the plaque-forming unit (PFU) assay [31]. Briefly, serial dilutions of extracted organs were prepared in infection medium (MEM containing 2% FCS) and used to infect Vero E6 monolayers in duplicates (200 µL/well). Plates were incubated for 1 h at 37 °C to allow viral adsorption. Then, 2 mL/well of overlay (MEM containing 2% FBS and 0.4% tragacanth (Merck, Israel)) was added to each well, and plates were incubated at 37 °C, 5% CO_2_, for 72 h. The medium was then aspirated, and the cells were fixed and stained with 1 mL/well of crystal violet solution (Biological Industries, Beit-Haemek, Israel). The number of plaques in each well was determined, and SARS-CoV-2 viral titer was calculated.

### 2.5. Quantitative Real-Time RT-PCR

Total RNA extracted from lungs and brains of mice infected with SARS-CoV-2 at 2 dpi and 5 dpi was used to measure differently expressed genes by qRT-PCR using the corresponding specific primers printed on 96-well plates (Custom TaqMan Array Plates, Applied Biosystems^TM^ (Waltham, MA, USA)) as previously described [32]. Briefly, cDNA was synthesized out of 1 µg of RNA using the Verso cDNA Synthesis Kit (Thermo Fisher Scientific, Waltham, MA, USA) according to the manufacturer’s instructions. Samples (500 ng of each cDNA sample per reaction) were subjected to qPCR with the TaqMan^®^ Fast Advanced Master Mix (7500 Real-Time PCR System, Applied Biosystems, Thermo Fisher Scientific). The following cycling conditions were applied: one cycle of incubation step (2 min/50 °C), 1 cycle of enzyme activation (20 s/95 °C) and subsequently 40 cycles of denaturation (3 s/95 °C) and elongation (30 s/60 °C). The housekeeping gene GAPDH was used to normalize the fold change of each gene compared to mock-infected control at the same time point and was calculated as ^∆∆^CT.

### 2.6. General Histopathology

For hematoxylin and eosin (H&E) general histology and viral detection by immunohistochemistry (IHC), mice (*n* = 5 per group) were anesthetized and then perfused transcardially with PBS. Lungs and brains were isolated and fixed in 4% paraformaldehyde at room temperature (RT) for 2 weeks. Lungs were spread horizontally in the cassette, and brains were dissected into four coronal sections showing the striatum, hippocampus, midbrain, cerebellum and brainstem. Serial sections, 4 µm thick, were performed using the Leica BOND-MAX system (Leica Biosystems Newcastle Ltd., Newcastle upon Tyne, UK), and selected sections were stained with H&E for light microscopy examination. The Leica Bond Polymer Refine Detection System Kit (DS-9800, Leica Biosystems Newcastle Ltd., UK) was used for detection and hematoxylin for counterstaining. For 3, 3′-diaminobenzidine (DAB) and H&E staining, pictures were taken using the Olympus microscope (BX60, Serial No. 7D04032) equipped with the microscope’s camera (Olympus DP73, Serial No. OH05504) at objective magnifications of ×4, ×10 and ×20. For immunofluorescent staining, images were acquired using the panoramic MIDI II slide scanner (DHISTEC3) at an objective magnification of ×1.3.

### 2.7. SARS-CoV-2 Immunohistochemical Staining

Immunolabeling of SARS-CoV-2 was performed as previously described [31]. Briefly, paraffin sections were incubated with rabbit SARS-CoV-2 primary antibody (in-house preparation of rabbit polyclonal anti-RBD) diluted 1:800 (for DAB staining) or 1:200 (for immunofluorescent staining) in antibody cocktail solution. For DAB immunohistochemical staining, a goat anti-rabbit horseradish peroxidase (HRP) polymer was applied for 10 min (RT), followed by incubation for 5 min with DAB. For immunofluorescent staining, slides were incubated with anti-rabbit Alexa Fluor 488 secondary antibody (Molecular Probes, Burlington, ON, Canada) in antibody cocktail solution for 1 h at RT.

### 2.8. Histological Evaluation

Lung and brain histopathological severity score analysis was performed using a semiquantitative grading scale of 4 points, as follows: Grade 0—the tissue appears normal; Grade 1—minor pathological findings; Grade 2—mild pathological findings; Grade 3—moderate pathological findings; Grade 4—severe pathological findings [33]. The intensity of the immunostaining reaction for the SARS-CoV-2 antigen was analyzed using a semiquantitative grading scale of 5 points for the intensity and number of positive stained cells, per a ×20 field, as follows: Grade 0—no positive reaction; Grade 1—only few cells are immune positive (<5 cells); Grade 2—very mild immunoreaction (5–15 positive cells); Grade 3—mild immunoreaction (15–25 positive cells); Grade 4—moderate immune reaction (25–50 positive cells); Grade 5—marked immune reaction (>50 positive cells).

### 2.9. Percentage of Air Area Calculation

Morphometric analysis of H&E images for calculations of air space ratio was determined using brightness segmentation in MATLAB software version 2020a. A representative image of each animal in the affected area at the same magnification (×10) was taken. Color threshold parameters were determined and remained consistent throughout the analysis. Total area values were measured separately for air space and tissue. Percentage of air area was calculated for each image. A total of 5 animals per group were used.

### 2.10. Flow Cytometry

Lungs were harvested on the indicated days post infection and processed using the Gentle MACS machine (Miltenyi, Bergisch Glabach, North Rhine-Westphalia, Germany) according to the manufacturer protocol. Red blood cells were lysed using RBC lysis buffer (BD Biosciences, Franklin lakes, New Jersey, U.S.), and cell suspensions were washed in PBS and resuspended in flow buffer (PBS supplemented with 2% FBS and 0.05% NaN3). Staining for extracellular markers was performed using the antibodies listed below for 20 min at 4 °C before fixation in 3% paraformaldehyde for 30 min at RT. Samples were collected on an LSR-Fortessa flow cytometer (BD BioSciences), and data were analyzed using FlowJo software v10 (Tristar, CA, USA).

The following mAb clones were used for staining: CD3 (145-2C11), NK1.1 (PK136), CD11c (N418) and SiglecF (1RNM44N). All antibodies were purchased from Thermo Fisher (San Diego, CA, USA). Aqua Live/Dead cell stain (Thermo Fisher) was used for exclusion of dead cells.

## 3. Results

### 3.1. Poly(I:C) Treatment Protects Mice against a Lethal SARS-CoV-2 Infection

**Figure 1:**
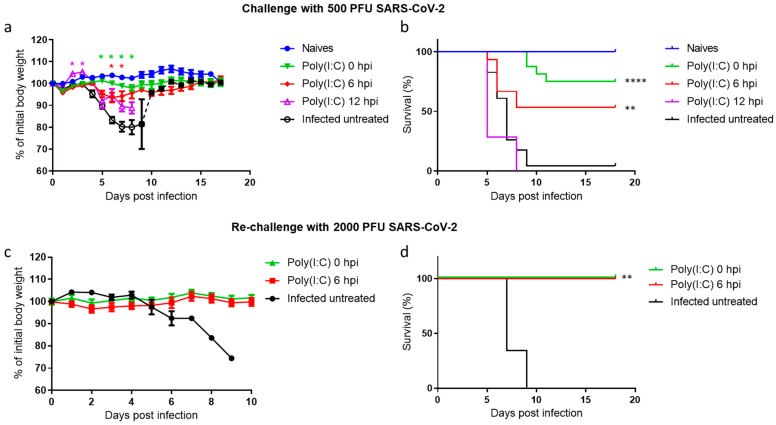
Poly(I:C) treatment protects SARS-CoV-2-infected mice. Body weight changes (**a**) and survival rates (**b**) of K18-hACE2 mice infected with SARS-CoV-2 (500 PFU i.n.). Number of animals per group: naïve, *n* = 12; Poly(I:C) 0 hpi, *n* = 23; Poly(I:C) 6 hpi, *n* = 8; Poly(I:C) 12 hpi, *n* = 7; Infected untreated, *n* = 23 (represents 3 independent experiments). The dashed line (**a**) represents 1 surviving mouse out of 23. Mice were rechallenged with 2000 PFU i.n. and monitored daily for body weight changes (**c**) and survival (**d**). Number of animals per group: Poly(I:C) 0 hpi, *n* = 5; Poly(I:C) 6 hpi, *n* = 4; Infected untreated, *n* = 3. Statistical significance was determined using the Holm–Sidak method * *p* < 0.005 (color coded; relative to infected untreated group) (**a**) or Mantel–Cox test ** *p* < 0.01. **** *p* < 0.0001 (**b**,**d**).

The therapeutic effect of Poly(I:C) against a lethal dose of SARS-CoV-2 was tested with the transgenic K18-hACE2 mouse model [34]. Mice were infected by intranasal (i.n.) instillation with 500 PFU of SARS-CoV-2 and treated i.n. with Poly(I:C) either at the time of infection (0 h post infection (hpi)), 6 or 12 hpi. Body weight changes (Figure 1a) and mortality (Figure 1b) were daily monitored for 17 days. Infected untreated mice started losing weight at 3 dpi and succumbed to infection 3–6 days later. However, mice infected and concomitantly treated with Poly(I:C) (0 hpi) displayed only minor body weight loss, resulting in 83% protection. Similarly, 6 hpi treatment was accompanied by a slight body weight loss with a survival rate of 50%. However, at 12 hpi, Poly(I:C) treatment did not result in beneficial effects on morbidity or survival rate compared with infected untreated mice. These results demonstrate that i.n. Poly(I:C) treatment is an efficient therapeutic intervention if given at early stages of the disease.

To check whether Poly(I:C)-treated mice that survived SARS-CoV-2 infection acquired long-term protective immunity, we rechallenged the animals one month following recovery from the first infection with a higher dose of 2000 PFU. While all infected untreated mice were morbid and died within 7–9 days post infection, all infected and Poly(I:C)-treated mice at either 0 or 6 hpi survived reinfection and did not lose weight (Figure 1c,d).

To further characterize the therapeutic window of Poly(I:C), we evaluated its prophylactic effect 6 h before SARS-CoV-2 infection (hbi). Poly(I:C) pretreatment ameliorated disease manifestations showed slightly improved survival rates compared to untreated mice that were morbid and succumbed to infection (Supplementary Figure S1). Based on these results, it is concluded that the protective time window of Poly(I:C) in this experimental in vivo system ranges from 6 hbi up to 6 hpi.

### 3.2. Poly(I:C) Treatment Reduces Viral Loads in Nasal Turbinates, Lungs and Brains

**Figure 2:**
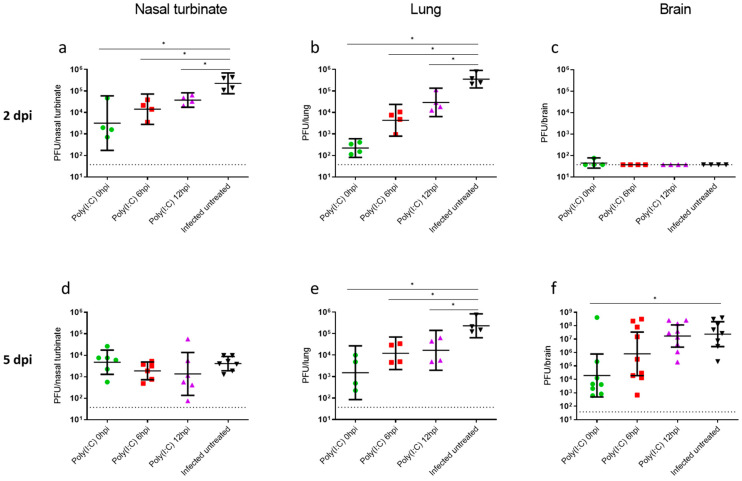
Poly(I:C) treatment reduces SARS-CoV-2 viral load. Virus titers in nasal turbinates, lungs and brains were measured at 2 (**a**–**c**) and 5 dpi (**d**–**f**) (*n* = 4 per group except nasal turbinates at 5 dpi, *n* = 6–7, and brain at 5 dpi, *n* = 8–9). Limit of detection (LOD): 37.5 PFU/organ. Data presented as geometric mean values with 95% CI. Statistical significance analysis was performed by using the Mann–Whitney test between groups. * *p* < 0.05.

To further characterize the therapeutic effect of Poly(I:C), we measured the viral loads in the nasal turbinates, lungs and brains of infected animals. Mice were infected by i.n. instillation with 500 PFU and treated with Poly(I:C) at the indicated time points or left untreated (Figure 2). Two or 5 dpi, mice were sacrificed, and their nasal turbinates, lungs and brains were collected and processed. Viral loads in the nasal turbinates and lungs at 2 dpi in infected untreated mice reached geometric mean titers (GMTs) of 2.3 × 10^5^ and 3.5 × 10^5^ PFU per organ, respectively. Poly(I:C) treatment resulted in a significant reduction in viral load in nasal turbinates to 3.2 × 10^3^, 1.4 × 10^4^ or 3.8 × 10^4^ PFU at 0, 6 and 12 hpi, respectively (Figure 2a). In lungs, the effect of the Poly(I:C) treatment following infection was even more pronounced; the viral titers were reduced to 2.2 × 10^2^, 4.3 × 10^3^ and 2.9 × 10^4^ PFU per lung at 0, 6 and 12 hpi, respectively (Figure 2b). The observed significant reduction in viral loads at both nasal turbinates and lungs following early Poly(I:C) treatments was in accordance with the significant improvement in their survival rates. However, Poly(I:C) treatment at 12 hpi resulted in a mild yet significant lung viral load reduction to 2.9 × 10^4^, which was no longer protective (compare Figure 2b with Figure 1b, respectively), suggesting that at this time point (12 hpi), other mechanisms than a reduction in viral load at the nasal turbinates, and/or the lungs determine the infection outcome. In brains, however, at 2 dpi, no viable virus could be detected (Figure 2c).

As the disease proceeded, viral loads at 5 dpi in the nasal turbinates were not significantly different between the treated groups in comparison to the infected untreated group, suggesting that the beneficial effect of Poly(I:C) in the nasal turbinates was limited to early stages of infection. In lungs, however, the Poly(I:C) effects on viral loads were more pronounced, resulting in about 1 log reduction (Figure 2e). Thus, further analysis on the effect of Poly(I:C) on the respiratory system was focused on lungs, representing the main site of infection.

While viral loads in brains were undetectable at 2 dpi in all treated or untreated mice, at 5 dpi, the viral load in untreated mice increased dramatically, reaching an average of 2.3 × 10^7^ PFU per brain (Figure 2f). Poly(I:C) treatment at the initial phase of infection (0 hpi) resulted in a significant reduction in brain viral loads by 3–5 orders of magnitude in 7 out of 8 mice, in accordance with the 83% survival rate obtained. Similarly, 5 out of 9 mice (55%) treated at 6 hpi resulted in a reduction of 3–5 logs, in line with the observed 50% survival rate. However, no reduction was observed in the Poly(I:C)-treated group at 12 hpi, similarly to the levels of infected untreated mice (Figure 2f).

### 3.3. Poly(I:C) Alleviates Lung Damage and Reduces SARS-CoV-2 Antigen in Both Lungs and Brains

The observed high viral loads in lungs and brains, and their significant reduction upon Poly(I:C) treatment, prompted us to examine the histopathological changes and the SARS-CoV-2 distribution pattern in these tissues. Based on previous studies [35,36], lung damage following SARS-CoV-2 infection was most pronounced at late stages of the disease, from 4 dpi onwards. In addition, since no viral loads could be detected in brains at 2 dpi, we focused our histopathological analysis on 5 dpi. To that aim, the lungs and brains of infected untreated and Poly(I:C)-treated mice were dissected and stained with H&E and immunolabeled for SARS-CoV-2.

**Figure 3:**
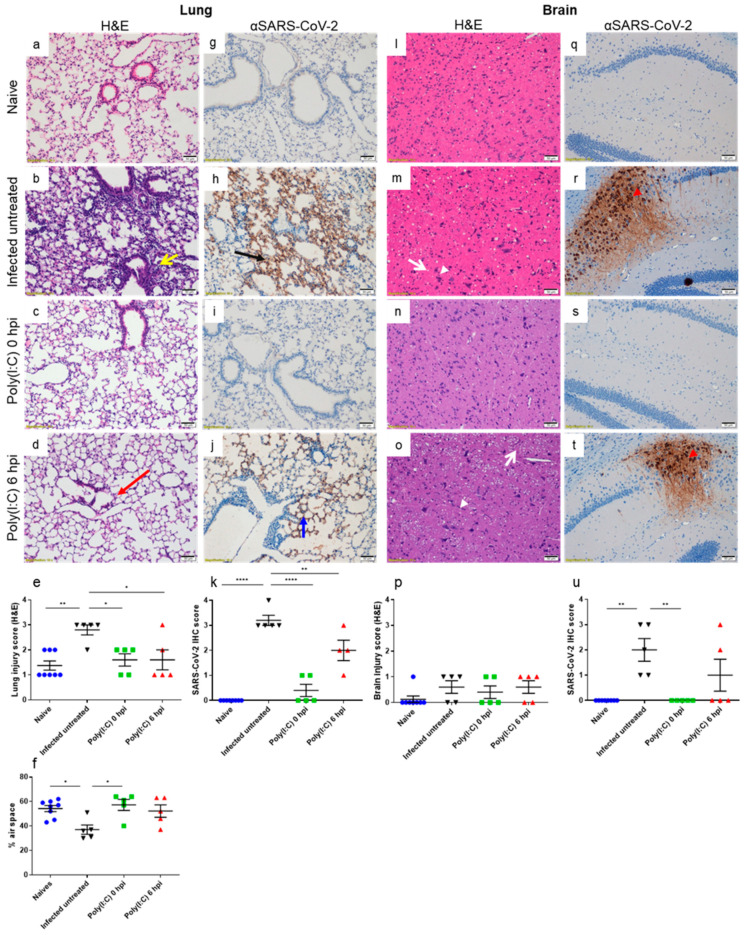
Poly(I:C) alleviates lung damage and reduces SARS-CoV-2 antigen in both lungs and brains. General histology (H&E) and SARS-CoV-2 DAB immunolabeling of lungs and brains of naïve, infected untreated, Poly(I:C) 0 and 6 hpi treated mice at 5 dpi. Lungs and brains were isolated and processed for paraffin embedding from naïve (**a**,**g**,**l**,**q**), infected untreated (**b**,**h**,**m**,**r**) and Poly(I:C)-treated mice at 0 hpi (**c**,**i**,**n**,**s**) and 6 hpi (**d**,**j**,**o**,**t**). Sections (4 µm) were taken for H&E staining (**a**–**d**,**l**–**o**) and SARS-CoV-2 DAB immunolabeling (**g**–**j**,**q**–**t**) (hematoxylin counterstaining—purple; SARS-CoV-2 immunolabeling—brown). Yellow arrow indicates moderate to severe bronchointerstitial pneumonia with fibrin deposition (**b**). Black arrow indicates moderate positive staining in alveolar septa (**h**); Red arrow indicates very mild cellular inflammation (**d**). Blue arrow indicates very mild positive staining (**j**). White arrowheads indicate very mild gliosis and white arrows indicate vacuolization (**m**,**o**). Red arrowheads indicate positive staining in hippocampal and cortical neurons (**r**,**t**). Scale bar = 50 µm; magnification = ×10. Histopathological damage severity score analysis of mice lungs (**e**) and brains (**p**) of naïve, infected untreated, Poly(I:C) treated at 0 and 6 hpi mice. Percentage of air space in lungs (**f**). Digital morphometric analysis of DAB immunohistochemical staining for SARS-CoV-2 in lungs (**k**) and brains (**u**) of naïve, infected untreated, Poly(I:C) treated at 0 and 6 hpi mice. Each group includes five animals. For each animal, 8–10 fields were imaged and analyzed. Data for (**e**,**f**,**k**,**p**,**u**) are presented as mean values  ±  SEM. Statistical analyses were performed by one-way ANOVA with Tukey’s multiple comparisons test, with * *p* < 0.05, ** *p* < 0.001, **** *p* < 0.00001.

As seen in Figure 3b, the lungs of infected untreated mice displayed moderate damage characterized by bronchointerstitial inflammation, including a cellular increase in alveolar septa (H&E staining). In contrast, treated mice displayed a similar morphological feature as observed in naïve mice (Figure 3a,c,d). Damage evaluation and percentage of air space were calculated (Figure 3e,f, respectively). Immunolabeling for the viral antigen in infected untreated mice was observed in different alveolar regions displaying a patchy distribution pattern (Figure 3h). Within the alveoli, pneumocytes and alveolar macrophages were positively stained. In bronchial epithelial cells, viral antigen was not detected. However, in Poly(I:C)-treated mice, a marked reduction in both antigen intensity and number of positive cells at 0 and 6 hpi was observed (SARS-CoV-2 labeling and semiquantitative analysis; Figure 3i–k, respectively).

In the brains of infected untreated mice, a very mild pathology was observed, including gliosis and demyelination (vacuolization in the white matter) in the affected regions (H&E staining; Figure 3m) as previously reported [37]. However, extensive labeling of SARS-CoV-2 was observed in all examined infected untreated mice (*n* = 5) throughout the brain, including the striatum, hippocampus, midbrain and cerebellum in the neurons and their processes (Figure 3r and Supplementary Figure S2). By contrast, Poly(I:C)-treated mice exhibited no or only minor positive immunolabeling at 0 or 6 hpi, respectively (Figure 3s,t), in agreement with reduced viral loads (Figure 2). Semiquantitative analyses of both brain damage and SARS-CoV-2 labeling were also calculated showing mild brain damage in all examined groups and a significant reduction in the number of SARS-CoV-2 positive cells in Poly(I:C) 0 hpi treated compared to infected untreated mice (Figure 3p,u).

### 3.4. Effect of Poly(I:C) Treatment on the Innate Immune Response Following SARS-CoV-2 Infection

In view of the reported immune subversion effects caused by SARS-CoV-2 [37] and the role of Poly(I:C) as an immune modulator, we aimed to characterize the inflammatory response induced by SARS-CoV-2 and the beneficial outcome of Poly(I:C) treatment by following the expression of 44 selected inflammatory-related genes. Lung and brain tissues were removed at 2 and 5 dpi, and the inflammatory-related gene expression profiles were analyzed by qRT-PCR. The genes tested were roughly divided into eight categories: cytokines (IFNα, IFNβ, IFNγ, IL-1α, IL-1β, IL-4, IL-6, TGFβ1 and TNFα); chemokines (CCL5, CCL8, CCL12 and CXCL10); IFN-induced proteins (IfI27l2a, IfI44, Ifit1, Ifit3, IRF7, Isg15 and STAT1); complement components (C1ra, C1rb, C1s1 and C3); costimulatory receptors (CD2, CD27, CD3e, CD3g, CD4, CD38, CD40, CD80, CD8a); antigen presentation markers (H2-EB1 and H2-K1); cell death effectors (FAS, FASl, Gzma, Gzmb, PRF1, ZBP1) and matrix metalloproteases (MMP3, MMP8, MMP9 and TIMP1).

**Figure 4:**
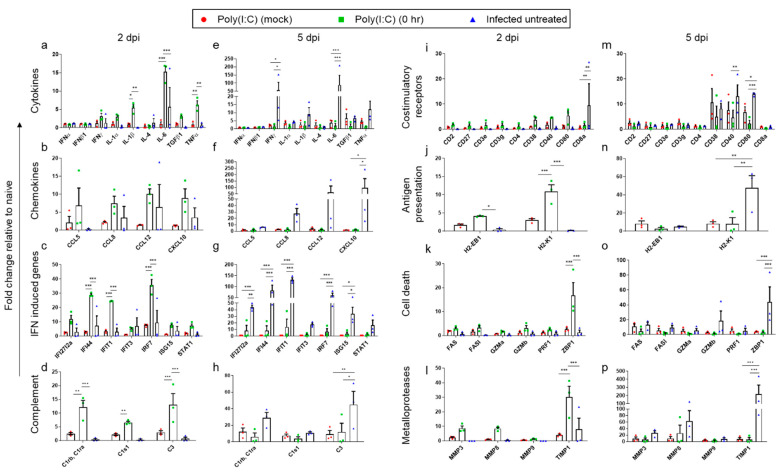
Poly(I:C) treatment induces a transient and synergistic innate immune response in the lungs of SARS-CoV-2 infected mice. RNA was isolated from the lungs of either Poly(I:C) mock, Poly(I:C) 0 hpi treated or infected untreated K18-hACE2 mice at 2 (**a**–**d**,**i**–**l**) and 5 dpi (**e**–**h**,**m**–**p**) and analyzed by real-time qRT-PCR. Each symbol represents one mouse (*n* = 3 per group). The *y*-axis represents the fold change compared with the naïve mice. The column height represents the mean. The error bars represent the SEM. Statistical significance analysis was performed using Two-way ANOVA with Tukey’s multiple comparisons test. * *p* < 0.05; ** *p* < 0.01; *** *p* < 0.001.

As seen in Figure 4a–d,i–l, at an early stage of the disease (2 dpi), Poly(I:C) treatment resulted in a sharp upregulation of gene expression of most gene transcripts examined in the lungs. On the other hand, neither Poly(I:C) treatment (mock) nor SARS-CoV-2 infection accounted for such an induction, suggesting that Poly(I:C) treatment in combination with viral infection results in a synergistic outcome. To further quantitate the synergistic inflammatory effect of Poly(I:C) and SARS-CoV-2, compared with the effect induced by either Poly(I:C) or SARS-CoV-2, the following considerations were made: genes were considered synergistically induced if their fold-change value (compared with naïve animals) in Poly(I:C) treatment in infected mice vs. the additive fold-change value of Poly(I:C) mock treatment and SARS-CoV-2 infection ratio is ≥1.2 [38]. Indeed, based on the above criterion, 77% (34 out of 44 genes) were synergistically induced (Supplementary Table S1). Among the most synergistically elevated genes were IL-1β, TNFα, MMP8, IFIT1 and ZBP1.

As the disease progressed (5 dpi; Figure 4e–h,m–p), a robust induction of 50–120-fold (compared to 2 dpi) in gene expression of IFNγ, IL-6, CCL12, CXCL10, IFI44, IFIT1, IRF7 and TIMP1 was observed in the lungs of infected untreated mice. By contrast, expression of the above-mentioned genes was significantly reduced in mice infected and concomitantly treated with Poly(I:C) to nearly basal levels. These observations are in accordance with the morbidity and mortality rates of infected untreated mice, reflecting the significant improvement gained by Poly(I:C) treatment on infected mice (Figure 1a,b).

**Figure 5:**
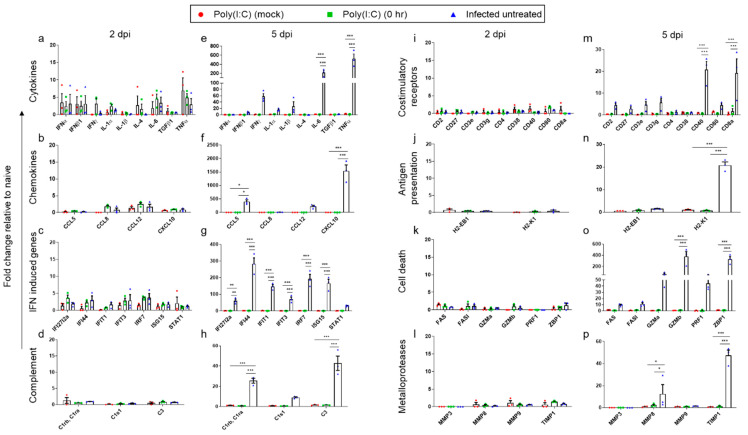
Poly(I:C) treatment prevents brain cytokine storm in SARS-CoV-2 infected mice. RNA was isolated from the brains of either Poly(I:C) mock, Poly(I:C) 0 hpi treated or infected untreated K18-hACE2 mice at 2 (**a**–**d**,**i**–**l**) and 5 dpi (**e**–**h**,**m**–**p**) and analyzed by real-time qRT-PCR. Each symbol represents one mouse (*n* = 3 per group). The *y*-axis represents the fold change compared with the naïve mice. The column height represents the mean. The error bars represent the SEM. Statistical significance analysis was performed using two-way ANOVA with Tukey’s multiple comparisons test. * *p* < 0.05; ** *p* < 0.01; *** *p* < 0.001.

Unlike the observed induction of multiple genes in the lungs early post infection (2 dpi), in brains, only slight induction of cytokines, chemokines and interferon-induced genes was identified in either infected and Poly(I:C)-treated mice or in the infected untreated animals (Figure 5a–d,i–l). These findings coincide with the undetectable brain viral load at 2 dpi (Figure 2c).

However, at 5 dpi, a sharp induction of inflammatory genes was observed in infected untreated mice brains (Figure 5e–h,m–p), but not in Poly(I:C)-treated mice, in accordance with the observed difference in viral load between these groups (Figure 2f). Some of the induced genes, such as IL-6, TNFα and CXCL10 (200-, 500- and 1500-fold change, respectively), are associated with severe brain cytokine storm [35], which may contribute to the poor prognosis of these animals.

To further characterize the differential immune response between lungs and brains following SARS-CoV-2 infection, we compared the expression of the inflammatory genes of infected untreated mice at 5 dpi between these two organs. As seen in Supplementary Table S2, 48% (21 out of 44) of the examined genes were upregulated by more than 2-fold in brains compared with lungs. The most pronounced upregulated genes were CCL5, TNFα, CD8a and Gzmb with values of 63.7-, 43.7-, 23.6- and 20.6-fold change, respectively. Conversely, 27% (12 out of 44) of the genes were upregulated by more than 2-fold in lungs (Supplementary Table S2). In this tissue, the most pronounced upregulated gene expressions were CD38, IL-4, MMP8, MMP9 and TIMP1 with values of 6.4-, 5.8-, 5-, 4.9- and 4.6-fold change, respectively. The remaining 11 genes were similarly expressed in both tissues.

### 3.5. Increased Levels of Alveolar Macrophages and NK Cells in the Lungs Following Poly(I:C) Treatment in SARS-CoV-2 Infected Mice

**Figure 6:**
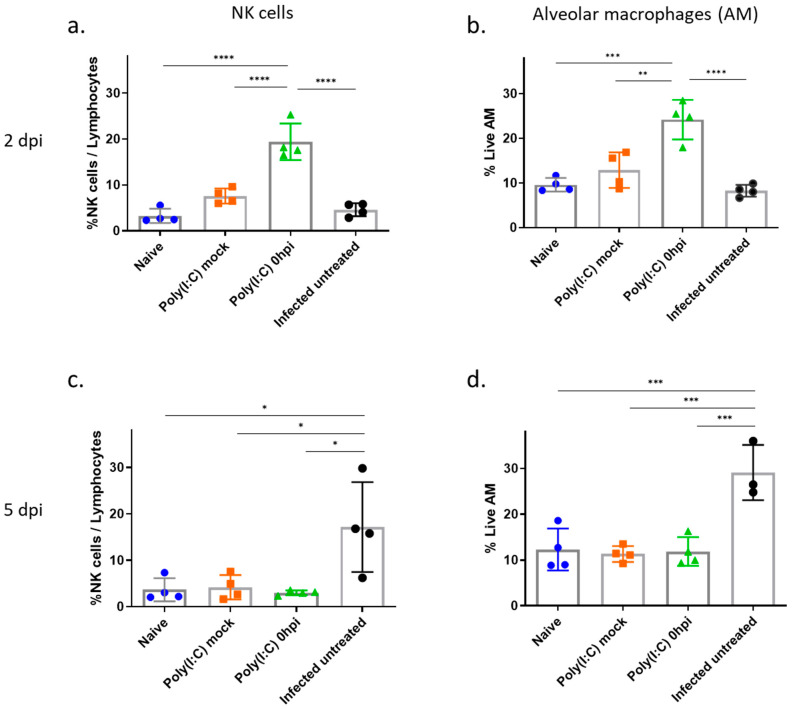
Increased levels of alveolar macrophages and NK cells in the lungs following Poly(I:C) treatment in SARS-CoV-2-infected mice. Lungs were harvested at 2 (**a**,**b**) or 5 (**c**,**d**) dpi, and the suspended cells were stained for NK and alveolar macrophages. The percentage of NK cells (NK1.1) and alveolar macrophages (CD11c+ MHCII+/− (I-A/I-E) SiglecF+) were calculated compared to total lymphocytes. Statistical significance analysis was performed by using one-way ANOVA. * *p* < 0.05; ** *p* < 0.01; *** *p* < 0.001, **** *p* < 0.0001.

The induction of immune-stimulatory genes by Poly(I:C) in the lungs of 2 dpi prompted us to follow the relative composition changes of alveolar macrophages and NK cells at the site of infection (lungs). As seen in Figure 6a,b, a significant increase in NK cells and macrophage populations was detected in infected and Poly(I:C)-treated mice. This effect was neither observed in infected untreated mice nor in mock Poly(I:C)-treated mice.

At a later stage of the disease (5 dpi), in the infected and Poly(I:C)-treated group, the early increase in NK cells and macrophages (observed at 2 dpi) returned to basal levels of naïve mice (Figure 6c,d). On the contrary, at this time point, infected untreated mice lungs displayed a significant enrichment of NK cells and macrophages, in line with the observed robust lungs cytokine storm.

## 4. Discussion

**Figure 7:**
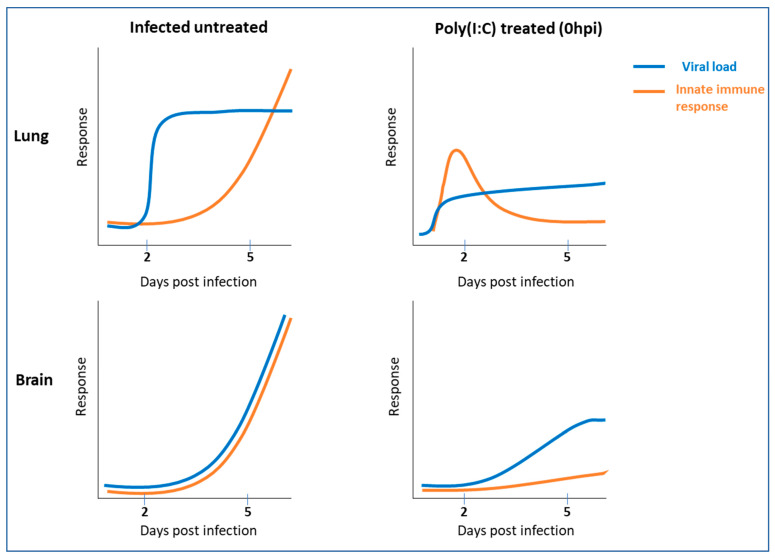
A model illustrating the therapeutic effect of Poly(I:C) following SARS-CoV-2 infection. In infected untreated mice, viral-induced silencing of the immune-related genes at early stages of the disease (2 dpi) is followed by a delayed yet excessive induction of innate immune-related genes (cytokine storm), high viral loads in the lung and brain (5 dpi) and death. On the contrary, intranasal Poly(I:C) treatment induces an early transient and controlled upregulation of the innate immune-related genes in the lungs (2 dpi), resulting in reduced lung (2 and 5 dpi) and brain (5 dpi) viral loads, prevention of cytokine storm and protection.

As the first line of defense, the innate immune system should react promptly and effectively to cope with pathogen invasion and its subsequent proliferation. In this study, we demonstrate that a single local treatment of the lungs with the TLR3/MDA5 synthetic agonist Poly(I:C) concomitantly with SARS-CoV-2 infection induces a transient upregulation of innate immune response, resulting in reduced viral load, alleviated morbidity and improved survival rates. On the other hand, in untreated mice, silencing of the immune-related genes in the lung at early stages of the disease was followed by a delayed yet robust induction of similar proinflammatory genes, massive viral replication, body weight loss and death (Figure 7).

SARS-CoV-2 is recognized by the PRRs MDA5 and NOD1, enabling an antiviral onset of the immune response. However, previous studies have shown that SARS-CoV-2 induces a delayed IFN-I response [13,39]. In addition, the virus has developed mechanisms for evading its recognition by the cellular PRRs. For example, the viral M protein [40], as well as the nonstructural protein 3 and 8 (nsp3, nsp8), can interact and suppress MDA5 activation [41,42]. In addition, Liu et al. recently demonstrated that MDA5 is antagonized by the SARS-CoV-2 papain-like protease [43]. In accordance with these findings, we demonstrate a robust increase in lung viral loads at 2 dpi, which is not followed by a concomitant induction of innate immune-related genes. By applying a PRR agonist such as the MDA5/TLR3 agonist Poly(I:C), the innate immune inhibitory effect of the virus can be overruled if applied locally at an early stage during infection. This was demonstrated by the intense favorable upregulation of the innate immune-related genes in lungs at 2 dpi, resulting in viral load reduction and 83% survival. A similar approach has been previously demonstrated by applying the PRR STING agonist, gaining full protection against SARS-CoV-2 infection [44].

The central role of PRRs in coping with COVID-19 has been recently suggested. For example, inborn genetic polymorphisms in TLR3-dependent IFN I signaling pathways were associated with COVID-19 life-threatening pneumonia [45]. Another study argued that the low percentage of COVID-19-hospitalized patients under the age of 18 is correlated with higher expression of MDA5 in their nasal turbinates compared to adults, resulting in an immediate response to infection [46]. Indeed, we demonstrated here that activation of both TLR3 and MDA5 pathways by Poly(I:C) protected mice from SARS-CoV-2 infection. In addition, the availability of a broad range of PRR agonists, which are approved for treatment, improves the chance of success in a heterogenic population and reduces the risk of resistance following traditional directed antiviral antibodies or drugs against various viruses [47].

Poly(I:C) is recognized by both endosomal TLR3 and cytosolic MDA5 PRRs [19], while SARS-CoV-2 has been demonstrated to be recognized by the cytosolic MDA5 and NOD1 PRRs [39], as well as by TLR2 [48]. Triggering multiple PRRs, especially when located in different cell compartments, can result in a synergistic outcome compared with the response of a single PRR activation [49]. NODs and TLRs have been shown to act synergistically, suggesting that agonists of these two PRRs groups may be combined as a powerful treatment against viral infection [50,51]. Indeed, we observed a robust upregulation of genes that occurs only in the SARS-CoV-2-infected and Poly(I:C)-treated mice, but neither in Poly(I:C)-treated nor SARS-CoV-2-infected animals. Such a synergistic response was accompanied by morbidity alleviation and an improved survival rate. Whether the mechanism underlying this synergistic activation is indeed the result of a concomitant viral and synthetic pathogen-associated molecular patterns (PAMPs), which induce multiple PRRs such as NOD1 (SARS-CoV-2) and TLR3 (Poly(I:C)), remains to be elucidated.

We observed that similar gene profiles were induced in lungs by both Poly(I:C) treatment in infected mice early during infection (2 dpi) or in infected untreated mice at a later stage of infection (5 dpi). However, while in Poly(I:C)-treated mice upregulation was moderate and transient, the upregulation of genes in infected untreated mice was robust, resulting in cytokine storm, pathological damage and death. These results suggest that both the timing and the extent of innate immune-related molecule induction conferred by PRR agonists, such as Poly(I:C), are crucial for protection. Thus, single treatment, rather than repetitive, administered at an early stage of the disease may account for the prevention of lung and brain cytokine storms observed in infected untreated mice, providing protection against viral infection. Moreover, Poly(I:C)-treated mice that recovered from SARS-CoV-2 infection were protected following rechallenge with a much higher dose (2000 PFU). This suggests that these mice have acquired long-term immunity, enabling them to cope with future SARS-CoV-2 infections. The lack of morbidity signs and mortality indicates the protection of these mice; thus, they are unlikely to develop lung and brain cytokine storms upon reinfection.

The histopathological damage observed at 5 dpi in the lungs of infected untreated mice is in accordance with high viral loads and extensive antigen distribution in this tissue. In brains, however, although extremely high viral loads were detected, no major histopathological damage could be observed. In addition, by comparing the expression profile of the induced gene-related innate immune system in brains vs. lungs upon infection, we show that the cytokine storm in the brain, which was previously reported in this system [35], surpasses considerably that of the lungs. For example, the expression profile of inflammatory response genes, such as TNFα and IL-6, two primary contributors to cytokine storms [52], as well as IFNγ, IRF7, IFI-44, Gzmb, ZBP1, Isg15, CCL5, CCL12 and CXCL10, were 3–60-fold higher in the brains compared with the lungs of infected untreated mice. These results are in line with neurological symptoms reported in more than 30% of infected patients [53]. Indeed, the apparent extreme brain reaction in this sensitive K18-hACE2 transgenic mice model of SARS-CoV-2 might not accurately represent the “real world”, since COVID-19 in humans is primarily considered a pulmonary disease. However, accumulating data on long COVID-19 neurological disorders even following full recovery from the disease may support the relevance and usefulness of this model.

It is well established that during aging, the immune system accumulates age-associated defects by compromising the innate immune cell function, as well as by signal transduction pathways, including TLRs [54]. Accordingly, old-aged mice were found to be much more susceptible to SARS-CoV-1 infection compared with young mice [20]. In this study, the authors also demonstrated the potential benefit of Poly(I:C) prophylactic intervention against SARS-CoV-1 in aged mice. Prophylactic i.n. treatment with Poly(I:C), 6 h before infection, resulted in the upregulation of various proinflammatory cytokines, enhanced T cell response and reduced viral load in the lungs, resulting in 100% mice protection. These results are in line with our data, further supporting the beneficial effect of Poly(I:C) on the innate immune system, resulting in mice protection. The differences, however, in the therapeutic time windows observed between the studies may be attributed to the differences in virus and mouse models. It is conceivable therefore that the treatment of old-aged mice will result in similar beneficial effects. However, the precise dose of Poly(I:C) may require few adjustments.

The rationale of using i.n instillation of Poly(I:C) is that COVID-19 is a pulmonary disease. We thus assumed that targeting the site of infection (and not systemically) and probably induction of mucosal immunity would be more efficient, especially in this mouse model, which displays a narrow window for therapeutic intervention. In addition, a safety study of i.n. administration of Poly(I:C) on humans was conducted as a potential therapeutic approach against various respiratory viral infections (https://clinicaltrials.gov/ct2/show/NCT00646152 (accessed on 12 January 2022)), pointing to the relevance of this route of administration in humans as well. Another important issue is that systemic (but not intranasal) Poly(I:C) administration in mice resulted in diaphoresis and transient weight loss (around 5%) [20].

The K18-hACE2 transgenic mice represent a very sensitive disease model for COVID-19, resulting in high viral loads in both lungs and brains. Additionally, the duration of the disease is much shorter in this mouse model, and the lethality rate is substantially higher than that observed with COVID-19 patients. Despite the differences between the human disease and the mouse model disease, it is broadly accepted as a reliable and robust small animal model for preclinical therapeutic studies on present and newly emerging SARS-CoV-2 variants. In that respect, it should be mentioned that clinical trials have recently been initiated by others for examining the therapeutic potential of Poly(I:C) against SARS-CoV-2 (https://clinicaltrials.gov/ct2/show/NCT04672291 (accessed on 12 January 2022)).

Altogether, we show here for the first time, to the best of our knowledge, that a synergistic but transient upregulation of innate immune-related genes can occur between a PRR agonist such as Poly(I:C) and a virus such as SARS-CoV-2. This novel paradigm highlights the importance of an efficient, local and early priming of the lungs by a PRR agonist, enabling a desired protective immune response.

## Supplementary Materials

The following supporting information can be downloaded at https://www.mdpi.com/article/10.3390/v14020189/s1, Figure S1: Prophylactic effect of Poly(I:C) following SARS-CoV-2 infection, Figure S2: Poly(I:C) reduces SARS-CoV-2 antigen in the brain, Table S1: Synergistic effect of Poly(I:C) in SARS-CoV-2 infected mice, Table S2: Comparison between the inflammatory response of lungs and brains following SARS-CoV-2 infection.
