# Supplementary material for: Induction of Innate Immune Response by TLR3 Agonist Protects Mice against SARS-CoV-2 Infection
Source: Viruses. 2022 Jan 19;14(2):189. doi: 10.3390/v14020189 (PMC8878863; doi:10.3390/v14020189)
Supplement: Supplementary file 1 [file viruses-14-00189-s001.zip › viruses-1535539-supplementary.pdf]

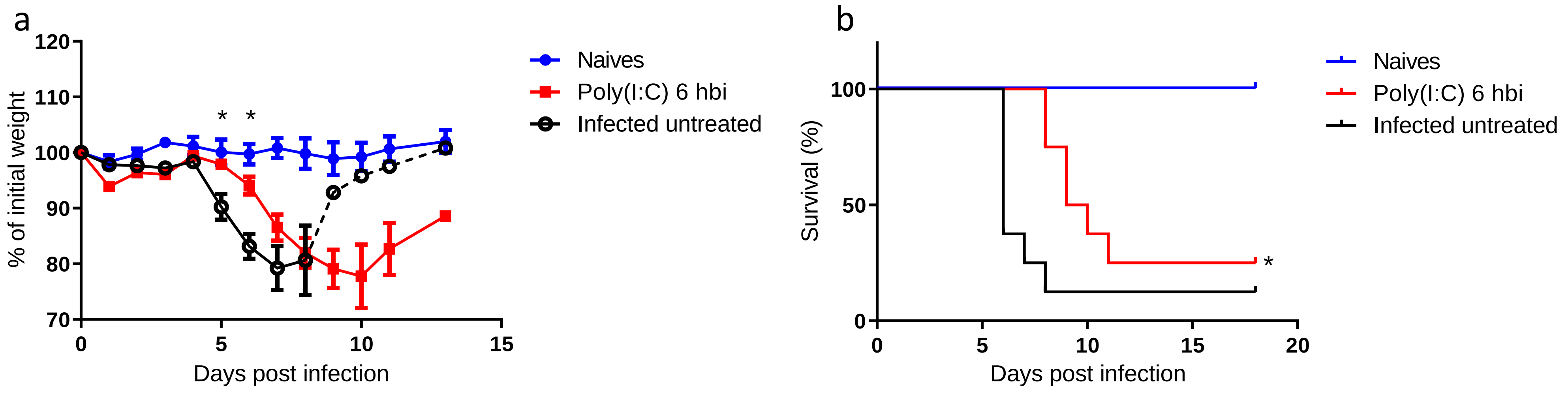

Figure S1. Prophylactic effect of Poly(I:C) following SARS-CoV-2 infection. Body weight changes (a) and mortality (b) of K18-hACE2 mice treated with 1.5mg/kg (i.n.) of Poly(I:C) 6 hours before infection with SARS-CoV-2 (500 PFU i.n.). Number of animals per group: naive n=3, Poly(I:C) 6 hbi n=9, Infected untreated n=9. Statistical significance was determined using the Holm-Sidak method as compared to infected untreated group *p<0.005 (a) or Mantel-Cox test *p<0.05.

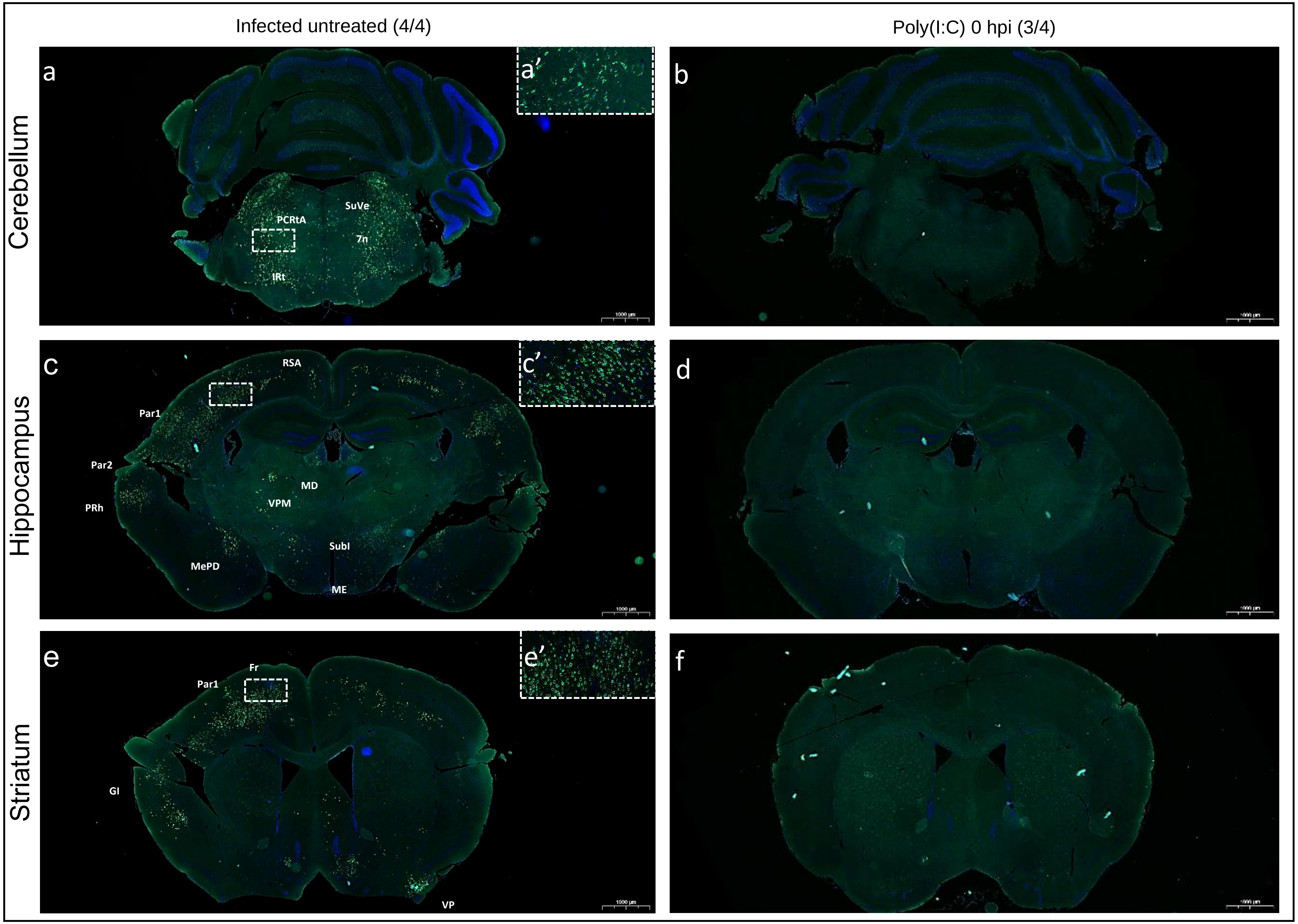
Infected untreated (4/4)
Poly(I:C) 0 hpi (3/4)
Cerebellum
Hippocampus
Striatum
a
a’
b
c
c’
d
e
e’
f
SuVe
PCRtA
7n
IRt
RSA
Par1
Par2
PRh
MD
VPM
SubI
MePD
ME
Fr
GI
VP

Figure S2. Histopathological brain analysis following Poly(I:C) treatment. SARS-CoV-2 immunolabeling of infected untreated and Poly(I:C) 0 hpi treated mice brains at 5 dpi. Brains were isolated and processed for paraffin embedding from infected untreated (**a**, **c, e**) and Poly(I:C) 0 hpi treated groups (**b**, **d, f)**. Scale bar= 1000µm. n=4 per group. PCRtA - parvocellular reticular nucleus alpha; IRt – intermediate reticular nucelus; SuVe – superior vestibular nucleus; VPM – ventral posteromedial thalamic nucleus; MePD – Medial amygdala nucleus posterodorsal; MD – mediodorsal thalamic nucleus. ME – median eminence; SuBI – Subincertal nucleus; PRh – perirhinal cortex; RSA – Retrosplenial agranular cortex; Par1 – parietal cortex area 1; Par2 – parietal cortex area 2; FR – frontal cortex; GI – Granular insular cortex; VP – ventral pallidum. **a', c'** and **e'** are enlargements of the boxes in a, c and e, respectively.

Table S1. Synergistic effect of Poly(I:C) in SARS-CoV-2 infected mice**.** The synergistic values of 44 inflammatory related genes of Poly(I:C) treated and infected mice lungs were calculated. Genes are considered synergistically elevated when the value is ≥1.2. Color scale bar indicating the fold change magnitude appears on the right.

Cytokines	
IFNα	0.4
IFNβ1	0.4
IFNγ	2.1
IL-1α	1.4
IL-1β	4.6
IL-4	0.2
IL-6	1.3
TGFβ1	1.6
TNFα	3.7

Chemokines	
CCL12	1.0
CCL5	2.5
CCL8	0.8
CXCL10	1.8

Complement	
C1rb, C1ra	2.0
C1s1	1.5
C3	1.9

Metalloproteases	
MMP3	1.8
MMP8	4.5
MMP9	1.3
TIMP1	1.4

Antigen presentation	
H2-EB1	1.5
H2-K1	2.1

Interferon related	
Ifi27l2a	1.6
Ifi44	2.4
IFIT1	3.0
IFIT3	0.6
IRF7	1.7
Isg15	1.3
STAT1	2.0

Costimulatory receptors	
CD2	1.6
CD27	2.1
CD3e	2.3
CD3g	1.6
CD4	2.2
CD38	1.5
CD40	1.5
CD80	2.4
CD8a	2.1

Cell death	
FAS	0.9
FASl	1.5
Gzma	0.9
Gzmb	0.9
PRF1	0.8
Zbp1	3.9

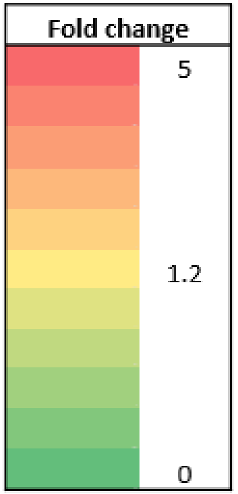

Table S2. Comparison between the inflammatory response of lungs and brains following SARS-CoV-2 infection. The fold change ratios of inflammatory related genes of infected untreated mice at 5 dpi that were upregulated by more than 1.2-fold in brain vs. lung (a) or lung vs. brain (b). Color scale bars indicating the fold change ratio magnitude appears on the right of each table.

Brain/Lung

a

	Fold change Brain/Lung
CCL5	63.7
TNFα	43.7
CD8a	23.6
Gzmb	20.6
CXCL10	15.8
Gzma	11.5
PRF1	8.8
Zbp1	7.5
IFNβ1	5.7
Slfn4	5.3
Isg15	4.9
IFIT3	4.1
IL-1a	3.8
CD3g	3.7
CCL12	3.6
Ifi44	3.4
IRF7	3.2
IL-1β	3.0
CD3e	2.6
IL-6	2.4
CD2	2.1
CD27	1.9
STAT1	1.9
CD40	1.6
Ifi27l2a	1.4
FASl	1.2

Fold change ratio	
65	
10	
1	

Lung/Brain

b

	Fold change Lung/Brain
CD38	6.4
IL-4	5.8
MMP8	5.0
MMP9	4.9
TIMP1	4.6
MKI67	3.4
H2-EB1	3.3
TGFb1	3.1
CD80	3.0
CCL8	2.3
H2-K1	2.3
FAS	1.4

Fold change ratio	
6.5	
1	
